# Mandibular Bone Loss: A Case Report of Idiopathic Osteolysis

**DOI:** 10.7759/cureus.91264

**Published:** 2025-08-29

**Authors:** Raina Shahani, Stuti Bhandari, Prashanth Rajaram, Niveditha N, Girish Giraddi, Sanjay S.C., Mahesh S Kanth, Chirag Lu

**Affiliations:** 1 Dentistry, DA Pandu Memorial RV Dental College, Bangalore, IND; 2 Internal Medicine, Kempegowda Institute of Medical Sciences, Bangalore, IND; 3 Oral and Maxillofacial Surgery, Vokkaligara Sangha Dental College and Hospital, Bangalore, IND; 4 Dentistry, Government Dental College and Research Institute Bangalore, Bangalore, IND; 5 Radiology, Kempegowda Institute of Medical Sciences, Bangalore, IND; 6 Rheumatology, ChanRe Rheumatology and Immunology Center and Research, Bangalore, IND; 7 Endocrinology, Kempegowda Institute of Medical Sciences, Bangalore, IND

**Keywords:** bone resorption in mandible, craniofacial pathology, gorham-stout disease, idiopathic osteolysis, mandibular bone loss, rare bone disease, vanishing bone

## Abstract

Mandibular bone loss can cause functional and aesthetic challenges. Idiopathic osteolysis, characterized by spontaneous and progressive bone resorption, may affect the craniofacial skeleton and often presents without pain or inflammation, leading to delayed diagnosis. This report presents a case of a 33-year-old female patient with mandibular bone loss exhibiting tooth mobility, pain, and difficulty chewing. Subsequently, the patient underwent mandibular reconstruction to restore anatomical integrity and improve functional capacity and psychosocial well-being. The case highlights the nonspecific presentation, which leads to late diagnosis and a complicated clinical course. With no universally accepted standard of care, management focuses on alleviating symptoms, preserving function, and preventing complications. We characterize this rare osteolytic condition through clinical, radiographic, and histopathological evaluation.

## Introduction

Mandibular bone loss is a pathological condition that can lead to significant clinical and cosmetic concerns. Bone resorption typically occurs due to identifiable causes such as infections, malignancies, or trauma. However, there are rare cases where bone resorption happens without a clear underlying reason. Osteolysis, which is characterized by localized bone resorption, is a common finding in radiological examinations. When it occurs spontaneously and progressively without an evident cause, it is classified as idiopathic osteolysis or disappearing bone disease [1].

Among the various forms of idiopathic osteolysis, Gorham-Stout syndrome stands out as a rare and complex condition with only 400 cases reported globally. Also known as vanishing or phantom bone disease and progressive osteolysis, it is histopathologically characterized by a proliferation of thin-walled vascular channels in bone [2].

While the etiopathogenesis remains unclear, theories suggest it may be related to vascular, lymphatic proliferation and enhanced osteoclast activity [3]. This condition can affect any gender, age, or race and commonly involves the pelvis, shoulders, and craniofacial area [4].

In the maxillofacial region, presentations may include facial asymmetry, tooth mobility, impaired occlusal function, pathological fracture, occlusal disorders, and dysfunctional mouth opening. Because pain and inflammation are often absent, late detection occurs, leading to a highly variable and unpredictable prognosis [5]. 

This case report mentions a 33-year-old female patient with marked mandibular bone resorption. We explore the diagnostic challenges and the importance of early diagnosis in managing rare bone disorders through detailed clinical, radiographic, macroscopic, and microscopic analysis. This report aims to give a clinical overview of this rapidly progressing and largely unknown disease of idiopathic osteolysis. 

## Case presentation

A 33-year-old female patient presented with chief complaints of pain in the lower jaw along with difficulty in chewing solid foods and mobility of the teeth of the lower jaw for three months. The patient had no history of deleterious habits, and her family history was unremarkable. The patient has no history of trauma or surgery. No history of steroid medication. 

On extraoral examination, the patient presented with no facial asymmetry. Deficiency was noted in the lower third of the face, indicating micrognathia. Intraoral examination revealed that the maxillary teeth, hard palate, tongue, and vestibular tissues appeared normal. Grade 2-3 mobility of mandibular teeth and pathological mesiolingual migration of mandibular molars 36,37 was observed. The patient had signs of malocclusion. On palpation, the absence of bone mass over the left and right parasymphysis, the inferior border and the symphysis region was apparent, and no tenderness or lymphadenopathy was observed. 

**Figure 1:**
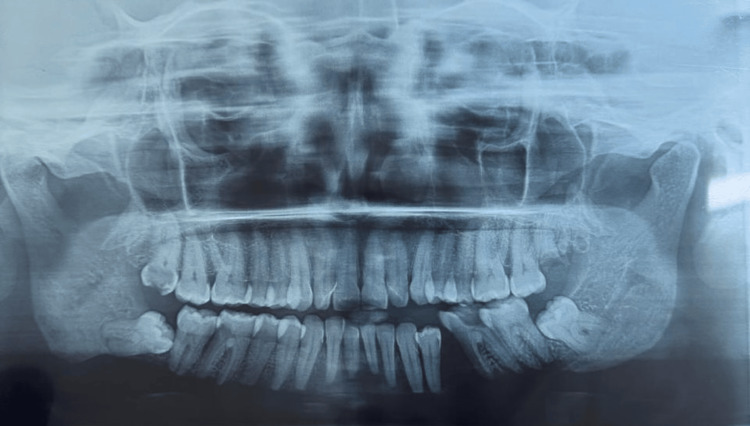
Panoramic radiograph image showing well-defined radiolucency extending from the right angle of the mandible below the roots of the right third molar to the body of the mandible below the left second molar, suggesting complete bilateral resorption of the lower border and mental process of the mandible. No resorption of roots is visible

**Figure 2:**
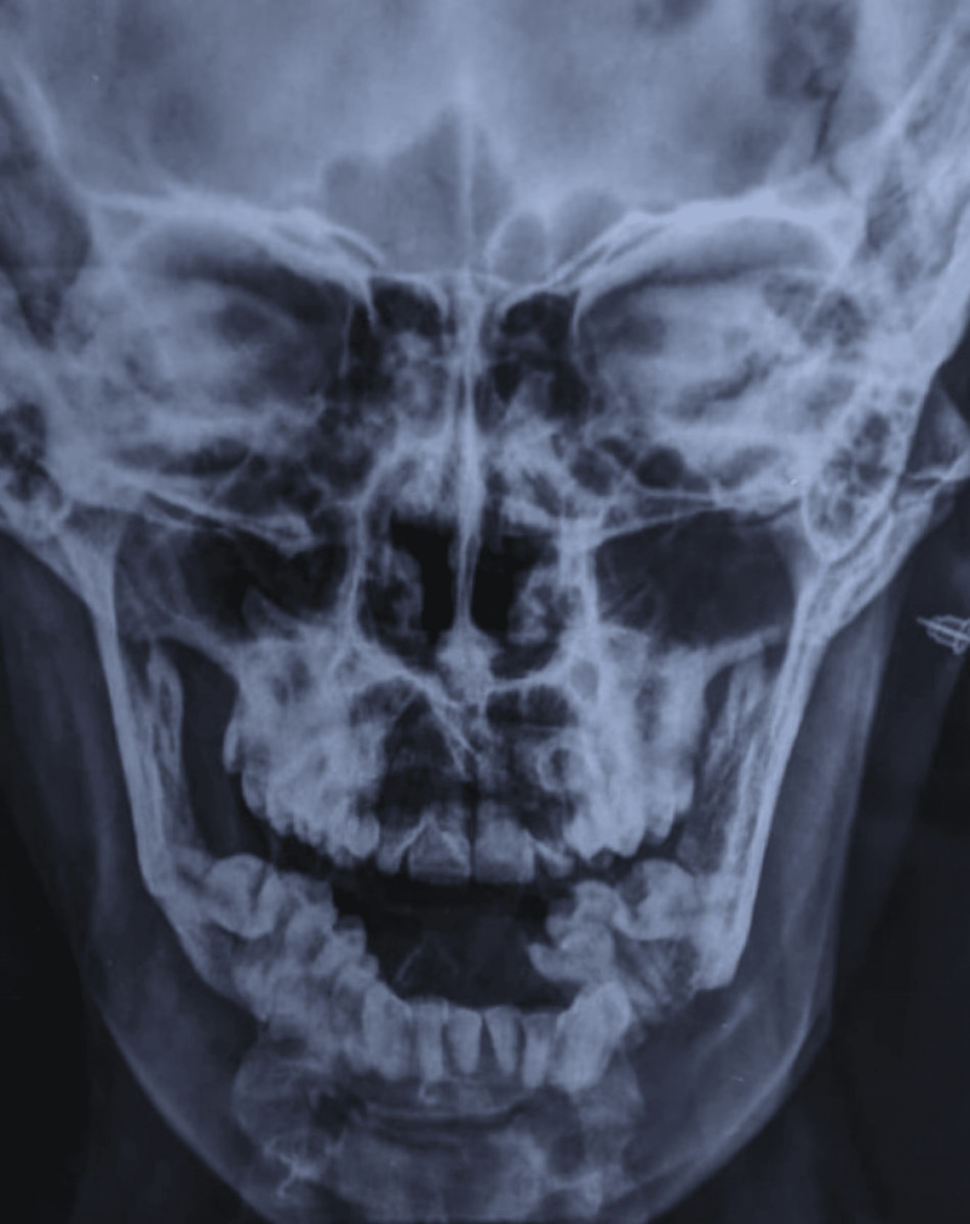
Plain X-ray of the skull and mandible, PA view PA view: posteroanterior view

**Figure 3:**
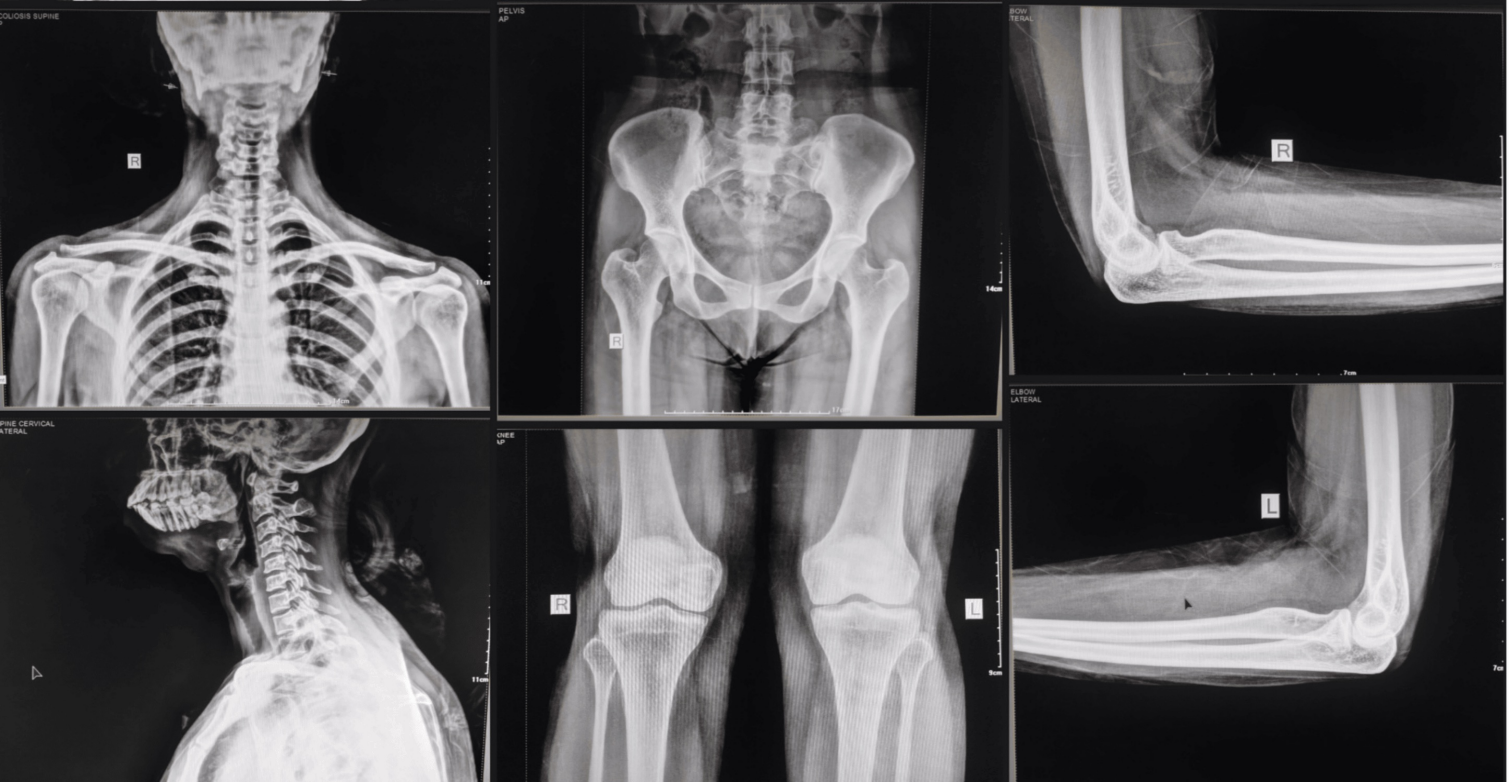
Plain chest X-ray AP view and lateral view. Plain X-ray of the right and left pelvis with the proximal part of the right and left femur AP view; plain X-ray of right and left knee showing distal part of femur and proximal part of the tibia and fibula AP view. Plain X-ray of the right and left elbow showing the distal part of the humerus and the proximal part of the radius and ulna, lateral view AP view: anteroposterior view; R: right; L: left

**Figure 4:**
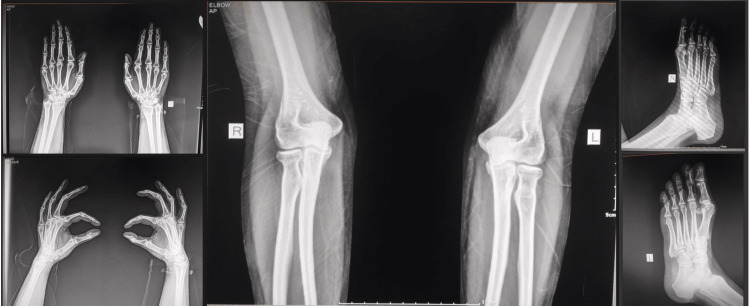
Plain X-ray of the right and left hand AP and lateral view. Plain X-ray of the right and left elbow showing the distal part of the humerus and proximal part of the radius and ulna AP view. Plain X-ray of the right and left foot with the distal end of the tibia and fibula in oblique view AP view: anteroposterior view; R: right; L: left

The patient underwent a series of investigations to determine the underlying pathology. Radiological imaging, including a panoramic radiograph (Figure 1) and plain X-ray of the skull and mandible (Figure 2), revealed mandibular loss as well as malocclusion, while the general skeletal survey (Figures 3, 4) appeared normal.

Routine investigations, including hematological parameters, metabolic profile, and rheumatological profile, (Tables 1-3, respectively), were unremarkable, except for mildly elevated erythrocyte sedimentation rate (ESR). A Mantoux/purified protein derivative (PPD) test (1TU/5PPD) was performed and yielded a negative result. Sputum analysis using real-time PCR for *Mycobacterium tuberculosis* complex also returned negative.

**Table 1:** Hematological investigations ESR: erythrocyte sedimentation rate; PCV: packed cell volume; MCV: mean corpuscular volume; MCH: mean corpuscular hemoglobin; MCHC: mean corpuscular hemoglobin concentration; WBC: white blood cell

Investigations	Results	Normal range
Hemoglobin percentage (g/dL)	12.9	12.0-15.0
ESR (mm/hr)	34	0-20
PCV (%)	40.3	36-46
Red blood cell count (million cells/cumm)	5.16	3.7-4.8
MCV (fl)	78.1	80-100
MCH (pg)	25.0	28-34
MCHC (g/dL)	31.9	32-36
Red cell distribution width (%)	15.8	11.5-14
Total WBC count (cells/cumm)	8500	4000-11000
Differential neutrophil count (%)	53.7	35-77
Differential lymphocyte count (%)	37.6	24-44
Differential monocyte count (%)	4.3	3-6
Differential eosinophil count (%)	3.7	0-3
Differential Basophil Count (%)	0.7	0-2
Platelet Count (lakhs/cumm)	4.46	1.5-4.5
Mean Platelet Volume (fl)	8.2	6.8-10.2
Peripheral Smear	Normocytic normochromic blood picture	

**Table 2:** Metabolic investigations PTH: parathyroid hormone; CRP: C-reactive protein; ALP: alkaline phosphatase

Investigations	Results	Normal range
Serum calcium (mg/dL)	9.8	8.6-10.0
Serum phosphorus (mg/dL)	3.4	2.5-4.5
Serum PTH (pg/mL)	58.10	12-88
Serum urea (mg/dL)	19	16.6-48.5
Serum creatinine (mg/dL)	0.69	Females: 0.5-0.9
CRP (mg/L)	3.06	<5
Serum ALP (U/L)	81	Females: 35-105

**Table 3:** Rheumatological investigations IFA: immunofluorescence assay; PR3-ANCA: proteinase-3-antineutrophil cytoplasmic antibody; MPO-ANCA: myeloperoxidase-antineutrophil cytoplasmic antibody; c-ANCA: cytoplasmic antineutrophil cytoplasmic antibody; p-ANCA: perinuclear antineutrophil cytoplasmic antibody

Investigations	Results	Normal range
Anti nuclear antibody by IFA	Negative	Negative
Rheumatoid factor (IU/mL)	7	0-20
PR3-ANCA c-ANCA (U/mL)	4.12	<12: Negative 12-18: Equivocal >18: Positive
MPO-ANCA p-ANCA (U/mL)	7.67	<12: Negative 12-18: Equivocal >18: Positive

Histological evaluation following an incisional biopsy was done. The microscopic examination of H&E-stained sections showed fibrous connective tissue without any epithelial lining. The lesional tissue, composed of connective tissue stroma, showed collagen fibers arranged haphazardly, interspersed with fibroblasts and fibrocytes. Thick-walled blood vessels, numerous endothelium-lined capillaries engorged with RBCs, nerve bundles, extravasated RBCs and mild inflammatory infiltrates were also evident in the connective tissue stroma. Nonvital bony trabeculae were noted in one area. No cellular atypia was observed. With clinical and histopathological correlation, the biopsy was suggestive of Gorham-Stout disease.

## Discussion

In this particular case, we report a middle-aged female who presented with bone loss whose hematological, metabolic, and rheumatological workup did not give a conclusive diagnosis. Given the clinical presentation, there are multiple differential diagnoses, each with overlapping symptoms. Some of the key possibilities include the following: Idiopathic osteolysis, proposed by Hardegger et al., has five subtypes. (1) Hereditary multicentric osteolysis with dominant transmission, whose predominant age of onset is between two and seven years, characterized by pain and swelling that usually begins in the hands and feet. (2) Carpo-tarsal osteolysis occurs over a few years, and progression ceases around adolescence. (3) Hereditary multicentric osteolysis with recessive transmission is similar to type one but is associated with generalized osteoporosis. (4) Nonhereditary multicentric osteolysis with nephropathy; this too occurs in childhood and manifests as the gradual disappearance of carpus with tarsal bone involvement but to a lesser degree and an association with proteinuria. (5) Gorham-Stout syndrome or vanishing bone disease, characterized by monocentric occurrence in any part of the skeletal tissue may start at any age. It has neither a hereditary pattern nor any associated nephropathy. Lastly, Winchester syndrome is transmitted in an autosomal recessive manner and is characterized by childhood carpo-tarsal osteolysis, contractures, skin lesions, short stature, corneal clouding, and osteoporosis, without associated nephropathy [6].

Of the abovementioned potential diagnoses, the one most consistent with the clinical and radiographic findings appears to be that of Gorham-Stout syndrome or vanishing bone disease. It is a rare disease of which only about 400 cases have been reported globally, of which 42 cases are of the maxillofacial region and 38 cases specifically involving the jaw. The earliest documented case of Gorham-Stout syndrome was reported by Romer in 1928 [1]. Herein, we present the first case reported in our institution. The exact pathology of this disease still hasn’t been completely understood. It is characterized by progressive resorption of bone due to nonneoplastic abnormal proliferation of blood vessels or lymphatic vessels and osteolysis caused by osteoclast proliferation or increased activity [3]. This theory is supported by the presence of immunohistochemical markers of lymphatic channels such as lymphatic vessel endothelial hyaluronan receptor 1 (LYVE-1) and podoplanin. Additionally, elevated levels of IL-6 seem to favor the involvement of osteoclasts [6]. Some of the lesser studied mechanisms proposed by de Keyser et al. noted that macrophages inhibit osteoblast function by producing tumor necrosis factor-alpha (TNFα) and that growth factors like vascular endothelial growth factor A (VEGF-A) and VEGF and platelet-derived growth factors play a role in lymphatic invasion [7]. An interesting point to note is that Gorham and Stout suggested that hyperemia, local changes in pH, and mechanical forces cause bone resorption and excluded any role played by osteoclasts [3]. Regardless of what might truly be the underlying etiopathology, what is evident is that the disease has an insidious and relatively pain-free onset, which advances until the affected bone is replaced by thin, fibrous tissue. 

Making a diagnosis of Gorham-Stout disease is mostly a diagnosis of exclusion. However, in 1983, Heffez et al. proposed an eight-point criterion for the diagnosis of Gorham-Stout disease, which is as follows: positive bone biopsy for angiomatous tissue, absence of cellular atypia, minimal or no osteoblastic response and absence of dystrophic calcification, evidence of local progressive osseous resorption, non-expansile and non-ulcerative lesion, absence of visceral involvement, osteolytic radiographic pattern, and negative hereditary, metabolic, neoplastic, immunologic, or infectious etiology [9].

The prognosis of Gorham-Stout syndrome is highly variable, ranging from minimal disability to even death if it progresses to affect vital skeletal components. Various other differential diagnoses can also include Paget’s disease of bone, melorheostosis, multicentric carpo-tarsal osteolysis syndrome (MCTO), and Torg syndrome [10]. Given the rarity and diagnostic complexity of idiopathic osteolysis, especially conditions like Gorham-Stout syndrome, thorough clinical and histopathological evaluation is crucial. 

As diagnosis is largely one of exclusion, awareness and application of established criteria can aid in identifying such cases. This report highlights the need for clinical vigilance and continued documentation to enhance understanding and guide the management of these rare bone disorders.

Treatment

Three primary treatment approaches are commonly utilized for this condition, each providing a unique strategy. These include a combination of medical, surgical, and radiological interventions. Surgical approaches focus on resecting the lesion and reconstruction with bone grafts or prostheses. However, this may only be carried out in the inactive phase, as it may lead to resorption of the graft in the active phase and a lack of recalcification of the osteolytic areas. Mandibular reconstruction is done with load-bearing long reconstruction plates covered by soft tissue. The primary drawback is the insufficient availability of adequate bone for proper fixation. In light of the patient's clinical status, surgical reconstruction was undertaken. Before surgery, the patient was advised to refrain from chewing hard or abrasive foods and was counselled on maintaining good oral hygiene. 

Other management strategies include radiation therapy, which is usually given in doses of 40-45 Gy in 2 Gy fractions and works on the mechanism of sclerosing blood vessels [3]. On the other hand, risks such as radiation-induced maldevelopment and malignant transformation in severe cases, and particularly orofacial adverse effects like osteonecrosis of the jaw, cannot be ignored. Lastly, other medical therapies include calcitonin and bisphosphonates. While vitamin D, androgens, calcium, steroids, and embolization have been used often, none of these treatment options has led to a permanent cure.

## Conclusions

In conclusion, this uncommon case is characterized by progressive bone resorption and dental instability, requiring a high degree of clinical suspicion for early diagnosis. While the treatment options remain limited and often focus on symptom management, ongoing advancements in research and multidisciplinary care offer hope for more effective therapeutic strategies in the future. Continued investigation into the underlying mechanisms of the condition is crucial to improving outcomes and enhancing the quality of life for affected individuals. 
